# Thinking about Social Media, Scientific Information, and Public Communication

**DOI:** 10.1128/msphere.00422-22

**Published:** 2022-09-19

**Authors:** Michael J. Imperiale, Arturo Casadevall

**Affiliations:** a Department of Microbiology and Immunology, University of Michigan, Ann Arbor, Michigan, USA; b Department of Molecular Microbiology and Immunology, Johns Hopkins School of Public Health, Baltimore, Maryland, USA

## EDITORIAL


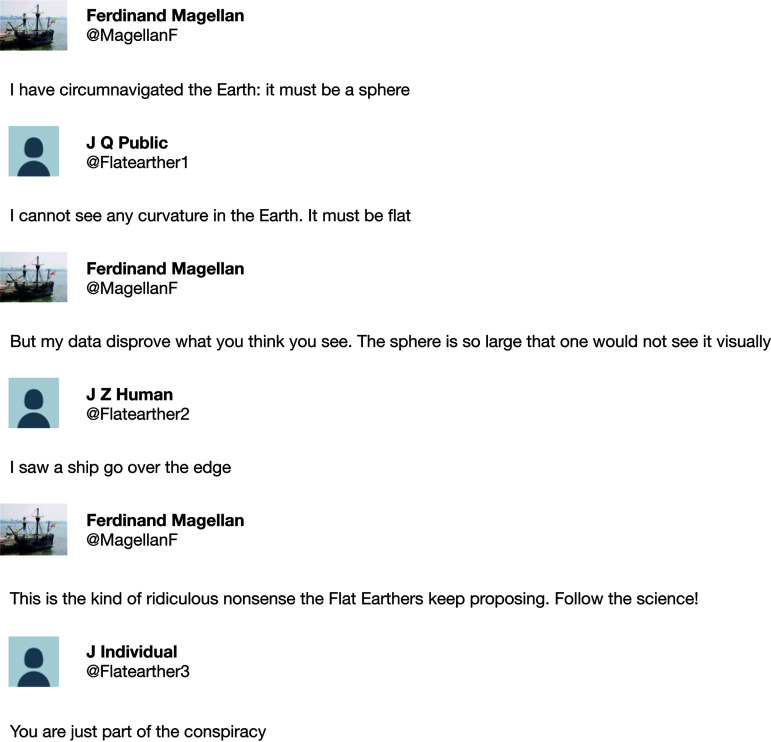


Obviously, this is a fictional Twitter thread: Magellan died 501 years ago. But the tenor of the thread is not false. Conversations on Twitter too often begin with opposing opinions and devolve into name calling and inuendo, and often go on much, much longer than this fictional example. In this editorial, we consider the advantages and disadvantages of scientific discourse in social media. We will argue that social media is often not well suited to having scientific discussions that depend on data driven arguments, particularly because of the diversity of individuals who may become engaged. In addition, when scientists inadvertently get pulled into the type of discourse shown above (or, worse, deliberately engage) they are doing a disservice to science and society. On the other hand, while social media is often an inadequate platform for scientific argumentation, it does provide a powerful mechanism for amplifying the reach and impact of scientific information, and scientists need to learn to use it effectively. As we were writing this piece, the *Chronicle of Higher Education* published a series of three opinion pieces on this same topic, aimed at a broader discussion of use of social media by academics writ large (1). We will focus on scientific discourse.

Scientific and technological advances have generally allowed for the betterment of society, especially in the areas of nutrition, material wealth, medicine and public health, communications, and travel, among others. Starting with the Industrial Revolution, new inventions and improvements to existing methods and tools have continuously enhanced our lives, from the development of the assembly line to the discovery of antibiotics to the Internet. While these advances were pursued to benefit society, it has been clear over the years that some technologies can also be used for harmful purposes, i.e., the so-called dual use dilemma. In recent years the dual use conversation has been focused on the potential of biological advances to bring benefits and harms, but dual use is an old concern previously encountered in other disciplines such as the physical sciences, as evident by the debates over harnessing the atom and nuclear energy. One classic example of the dual use dilemma was the discovery of the Haber-Bosch process for chemically fixing atmospheric nitrogen into ammonia in the early years of the 20th century, which allowed synthesis of both fertilizers for increased food production and munitions for warfare. We would make the argument that social media is not exactly like these other technologies in that there are not just the binary categories of beneficial and maleficent use: there is a third category, which we would characterize as “of dubious use,” that can be just as dangerous as misuse.

For the purposes of this essay, we are focusing on primarily on Twitter, but many of the concepts apply to other platforms that are currently in use. The common features of these platforms are their ubiquitous use; the opportunity for a dialog between or among users who often do not know one another; 24/7 availability; role as a sole (or nearly sole) source of information for many people; user anonymity and parody accounts, which can often enable mean-spiritedness, antagonism, trolling, and other abusive behaviors; and, with some notable topical exceptions, lack of restrictions on what can be posted.

The benefits of social media are many. Friends and family who live far away from one another can keep in touch. Legitimate news media outlets can push out breaking news and links to the full stories on their websites. Retailers can notify shoppers of sales. Government agencies can send out warnings such as Amber Alerts or severe weather watches. We ourselves have used social media to promote some of the manuscripts published in the journals of which we serve as Editors-in-Chief, as well as the work from our laboratories. For both authors, social media has sometimes been the first to inform us of important scientific developments and national news. In other words, these platforms can be the source of important information that can be disseminated quickly. But we know, of course, that social media can also be a platform for the distribution and amplification of inaccurate or incorrect information, personal attacks, and outright lies. Often, these falsehoods have been propagated as part of a political agenda, such as the attempts by Russia to influence the 2016 U.S. presidential election.

In considering the profound consequences of the 21^st^-century information revolution on our uncertain world, it is worth noting that the introduction of the printing press in the 15th century had a similar unsettling effect on the late medieval world. It has been argued that both the Protestant Reformation and the scientific revolution were outcomes of the introduction of the printing press in Europe, which disrupted societal structures to allow for new views to emerge (2). Hence, our field, science, emerged from an earlier information revolution that also engulfed Europe in 2 centuries of wars of religion, which brought untold suffering. We can only hope the information revolution in our time unfolds without comparable calamities. We cannot wholly understand the profound consequences of social media on our society because we are simultaneously experiencing them, and their full appreciation will require time and analysis of the present in a future historical context. Yet here we are, these times must be lived, and the question is how to optimize the power of social media while minimizing its debits.

This brings us to the topic at hand, namely, the appearance on social media of information and misinformation related to scientific topics. Two such topics that have received a lot of attention in recent years are vaccination and COVID-19, sometimes in a related manner. Experimental scientists and the public health community, along with organizations such as the CDC and WHO, have used social media to quickly spread emerging discoveries about SARS-CoV-2, and to provide updates on the development and use of therapeutics and vaccines. Others, however, have pushed the use of ineffective and sometime dangerous treatments such as bleach or ivermectin, and have peddled various unsubstantiated theories about COVID-19 vaccines, including how they are a means for the government or software companies to implant microchips that will be used to track our every move, or how they contain toxins that will enter our brains and cause lesions.

It is at the intersection of these two uses of social media where we have some concerns. As the fictional example at the beginning of this essay demonstrates, what begins as a legitimate discussion can all too easily turn into a back-and-forth barrage of snark and, sometimes, ad hominem attacks. Moreover, as more and more individuals join a thread, there is a tendency for tangential points to be made that can cause an almost exponential increase in posts that are not relevant to the original post, often leading into discussions that more reflect societal political polarizations than a useful discourse.

What can we, as scientists, do to avoid these pitfalls and, more importantly, how can we turn these platforms into more constructive means of educating the world about the scientific method, how we think about data, and what the data mean, rather than exacerbating factionalism? Here are some suggestions.

**Be aware of the context of social media.** As we have noted, social media reflects society as a whole. It represents the democratization, if you will, of the way information is communicated to the public at large. No longer are we dependent on a few broadcast television channels or towering news personalities like Walter Cronkite, Tom Brokaw, or Dan Rather to tell us everything we need to know in a 30 minute daily news show. If we wish to maintain our credibility as scientists, we must understand that what we say, where we say it, and how we say it matters, and that postings on the Internet are forever, which means that someone can always find a post from long ago and possibly use it out of context.

**Be humble.** We should always remember that much of the public does not understand the scientific method. Regrettably, this also applies to some scientists. We ought to be forthright when there are things about which we are not 100% certain. And, when we do misstate something, or when the scientific process works as it should and our knowledge evolves, we should be willing and able to admit that our previous notions have changed (or were wrong) and explain why in an understandable manner. Perhaps the most difficult aspect of science for the public is that all scientific knowledge is provisional, which means the knowledge base is constantly evolving. Consequently, opinions and recommendations will change over time, sometimes rapidly. Furthermore, all scientific knowledge comes with some degree of uncertainty, which clashes with the public need for unambiguous answers, especially during a crisis such as the COVID-19 pandemic. An example of this is the discussion of use of masks early in the pandemic. It was not clearly stated at the beginning of the pandemic that the use of masks by the public was being discouraged because there was a shortage, and health care providers and staff needed to be given priority access to the masks that were available. Furthermore, in the early days of the pandemic the efficacy of masking as a mechanism to prevent infection was uncertain until it became clear that the virus was transmitted by aerosols. Rather, what people heard was, “First you said don’t wear masks, then you said wear them.” How does one know what is truth, and whom to believe? As scientists, we take the answer to this question for granted, but many people just do not know. Many individuals get their information from social media posts, websites, or irreputable cable TV broadcasts, and do not have the ability to easily distinguish fact from fiction. It is for this reason that we deliberately call the above post from @Flatearther1 “legitimate”: it was reasonable to state that the Earth was flat, based on that person’s own observation and the prevailing views at the time. It is important to keep this societal context in mind because it is not just our followers on social media who will read what we write, but if they respond or retweet, it is also their followers, or the people who follow hashtags that we and others use. Making fun of or dismissing these individuals is not helpful, often only paints academics and scientists as “elites” who cannot be trusted, and leads to more polarization. Before we post, let’s put ourselves in their shoes.

**Be educators.** Many scientists at academic institutions teach as part of their positions. Even those of us who are at research institutes, industry, or government must present our work at conferences and seminars. We know the importance of clear communication and targeting what we say to the specific audience. If a concept is complicated, let’s find a way to make it understandable to the public at large if we are going to post it to a social media platform. Let’s remember that most people who see our posts may not be as knowledgeable as the students in the science classes with whom we interact daily. We know that social media platforms do not always facilitate this type of nuanced communication, with character limits and the like. But that does not mean that we cannot be creative: for example, we can produce short videos, or link to websites, that are understandable to the average person. Let’s make social media a platform to help lead the average person to an understanding of what we do and what the data mean…and do not mean.

**Be aware of your motivation.** What is your intent when engaging in social media? Is it to simply put information out there, or are you making an attempt to educate some group of people? If the latter, please consider that your audience may be that which you intended or may not be (see our earlier points about who sees posts). Are you advocating for a particular policy? If so, make that clear and acknowledge the pros and cons.

**Be scholarly.** When presenting scientific information, be scholarly in the sense of providing a reputable, understandable reference for statements made. This has two purposes. First, it gives the audience a mechanism for reading further and grounds whatever statement is made on the existing knowledge and literature in a given field. Second, it minimizes the likelihood that the statement made is incorrect since the mere act of looking up and checking a reference can enhance the accuracy of the text written. In this regard, articles in scientific journals may not be the best sources to cite because they may be too dense and filled with jargon for a nonscientist (or even a scientist in a different field) to comprehend. This can then lead to misinterpretations, causing additional opportunities for misinformation to spread.

**Be polite and considerate.** The terse nature of social media communications provides fertile ground for misunderstanding. Statements made in good faith can be misinterpreted and challenged. It is therefore best to pause and reflect before responding. This is especially important when a challenge to what we post is aggressive and confrontational. If the challenge is completely out of line it may be best not to respond at all as this will hopefully be apparent to other readers, and further confrontation may exacerbate the situation without changing any minds. Obviously, every challenge is different, and we cannot provide advice for all situations. If one chooses to respond, politeness and consideration are always good rules to follow. Sometimes it is simply better to let things go and not try to have the last word.

**Be cognizant of our obligation to society.** We would like to reiterate and expand on a point that we have made previously in other contexts. Let us remember why we are scientists: we want to understand the natural world around us and use that information to make it better. Who are the beneficiaries of what we do? The public. And who, for the most part, funds what we do? The public. Therefore, let’s show the public that we are focused on problems relevant to them and that we are trying to help them live better, healthier lives. That if they are unsure of something, they should be able to trust us to be truthful and transparent without being condescending and dismissive. That we want them to be able to count on us to be their allies, not a group of elite, disengaged individuals who are not even trying to understand how they view the world in which we all live.

Let us be clear: we understand fully that what we are asking for is not simple to accomplish. Our society is at a point at which constructive dialog can be difficult to achieve. Electronic means of communicating such as email and social media have put us in a mindset in which we often feel a need to respond rapidly, sometime to the detriment of being as thoughtful as possible. The past two pandemic years have put everyone on edge and lowered our collective tolerance. But we owe it to ourselves, as scientists who seek the truth and who make a concerted effort to help everyone lead a better life, one that is dependent on the discoveries we make each and every day, to strive to be a key part of the communities in which we live. We can be part of the solution rather than contributing to the chaos.
